# Perioperative Safety of Bilateral Internal Thoracic Artery Coronary Bypass in Elderly

**DOI:** 10.31083/j.rcm2401004

**Published:** 2023-01-03

**Authors:** Ryan Chaban, Ahmed Ghazy, Hendrik Treede

**Affiliations:** ^1^Department of Cardiovascular Surgery, University Hospital of Johannes Gutenberg University, 55131 Mainz, Germany

**Keywords:** cardiothoracic surgery, coronary artery bypass surgery, elderly patients, internal thoracic artery

## Abstract

**Background:**

The benefits of utilizing internal thoracic arteries (ITAs) in coronary bypass surgery are well-known. However, the safety of this practice in elderly patients needs to be proven.

**Methods:**

We studied all patients who are 75 years of age and older, who received at least one ITA graft while undergoing isolated, conventional (median sternotomy) coronary artery bypass graft surgery (CABG) between Jan 1st 2002 and Dec 31st 2020 (19 years). Emergent surgeries were excluded. Propensity score matching was used to reduce the patient selection effect. Study outcomes were 30-days mortality, and two sets of dependent intraoperative parameters and postoperative parameters.

**Results:**

A total of 1855 patients undergoing CABG was included, of which 1114 received a single left (s)ITA and 741 received combined left and right (d)ITA grafts. 519 pairs were matched. The decision for sITA or dITA was made individually. Thirty-days mortality was low and similar in both groups (sITA 3.3%; dITA 2.9%, *p* = 0.859). The incidence of sternal wound healing disorder was higher after dITA (3.3 vs 6.9%; *p *< 0.011), which had also a longer skin-to-skin operative time (181 vs 205 min; *p *< 0.0001). Re-thoracotomy rates were similar (4.6 vs 6.2%; *p* = 0.340). There were no significant differences in other secondary parameters.

**Conclusions:**

harvesting both ITAs in elderly patients is safe and feasible. However, it increases the risk of sternal wound healing disorders. Long term benefit still needs to be proven.

## 1. Introduction

The benefits of arterial conduits in coronary artery bypass grafting surgery (CABG) are well known [1, 2, 3]. Utilizing the internal thoracic (mammary) arteries (ITAs) for this purpose is highly recommended and improves long-term survival [1]. Harvesting both ITAs is well tolerated and associated with minimal side effects among younger and less morbid patients [4]. However, Long-term benefits and survival advantage are still a matter of ongoing debate [5, 6, 7].

Balancing the pros and cons of utilizing the internal thoracic arteries in the elderly and in patients with comorbidities is more complicated. Theoretically, as the pre-operative life expectancy shortens, the survival advantage of utilizing ITA grafts diminishes. A survival benefit for bilateral ITA harvesting in the elderly has not been confirmed [8]. The side effects of this practice become more relevant and less favorable for these patients. Complications, e.g., wound healing disorders and sternal instability, are seen at higher rates, especially in the presence of additional risk factors, e.g., obesity, female gender and comorbidities such as diabetes mellitus, chronic pulmonary disease, renal insufficiency. Hence, hesitancy still exists regarding the use of ITAs in CABG surgery in elderly patients [9].

The primary objective of this study is to evaluate the safety of harvesting both ITAs in the elderly by comparing the short-term results in patients who are 75 years of age and older who received either single left (sITA) or double (dITA) internal thoracic artery grafts.

## 2. Methods

### 2.1 Data Acquisition and Ethical Aspects

**Fig. 1.**
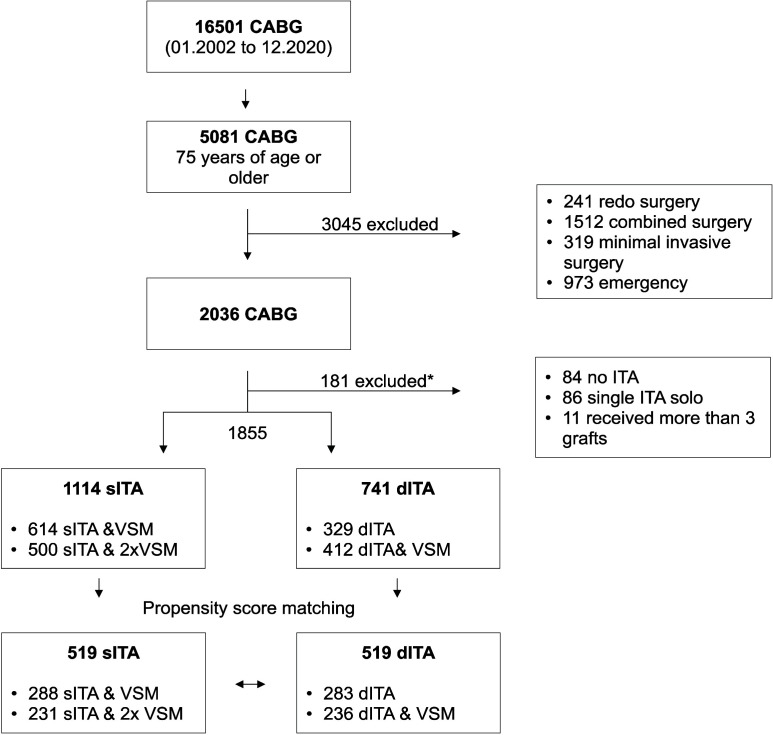
**Study design**. CABG, coronary artery bypass graft; ITA, internal thoracic artery; VSM, vena saphena magna; *, according to excluding criteria.

This study was conducted in accordance with the relevant guidelines for good clinical and good scientific practice defined by the 1964 Helsinki declaration and with approval of the university hospital of Mainz institutional board. Patient consent for anonymous use of their data in this retrospective study was obtained indirectly as a part of the hospital admission process. This is a retrospective single-center study conducted in the department of cardiothoracic and vascular surgery in the university hospital of Mainz. All documented primary (redo-surgeries were excluded), isolated (combined procedures, e.g., CABG + valve surgery were excluded), conventional (complete median sternotomy) CABG procedures, that were conducted between Jan 1st 2002 and Dec 31st 2020 (19 years) in patients who are 75 years of age and older and received at least one ITA graft, were included (Fig. 1). Excluding criteria were: emergent surgeries (defined as surgeries that were conducted within 24 hours of unplanned admission of cardiac patients or a myocardial infarction within 48 h before surgery), isolated single coronary artery CABG or CABG with more than 3 grafts (Fig. 1). The period of the study extended from the initiation of digital archiving in our department to the time of conception of the study. Patient data was collected and analyzed in a blinded fashion from the institutional database.

### 2.2 Surgical Technique

Surgical technique is standardized in our department with the classical steps of CABG. Median complete “cranio-caudal” sternotomy was carried out. Single left (sITA) or double (dITA) were harvested using a skeletonized or pedicle technique. Skeletonization was performed using an ultrasonic scalpel (Harmonic Synergy Blades ®, Ethicon GmbH, Norderstedt, Germany) to open the endothoracic fascia and dissect the ITA from the adjacent veins. The ITA branches were coagulated by applying the tip of the blade directly onto the vessel for 3–5 seconds. Hemostatic titanium clips were only used for major branches. For the pedicle technique, an electrical knife was used to harvest the ‘pedicled’ ITA with the surrounding veins, muscle and fascia, after closing off the branches with hemostatic clips.

**Fig. 2.**
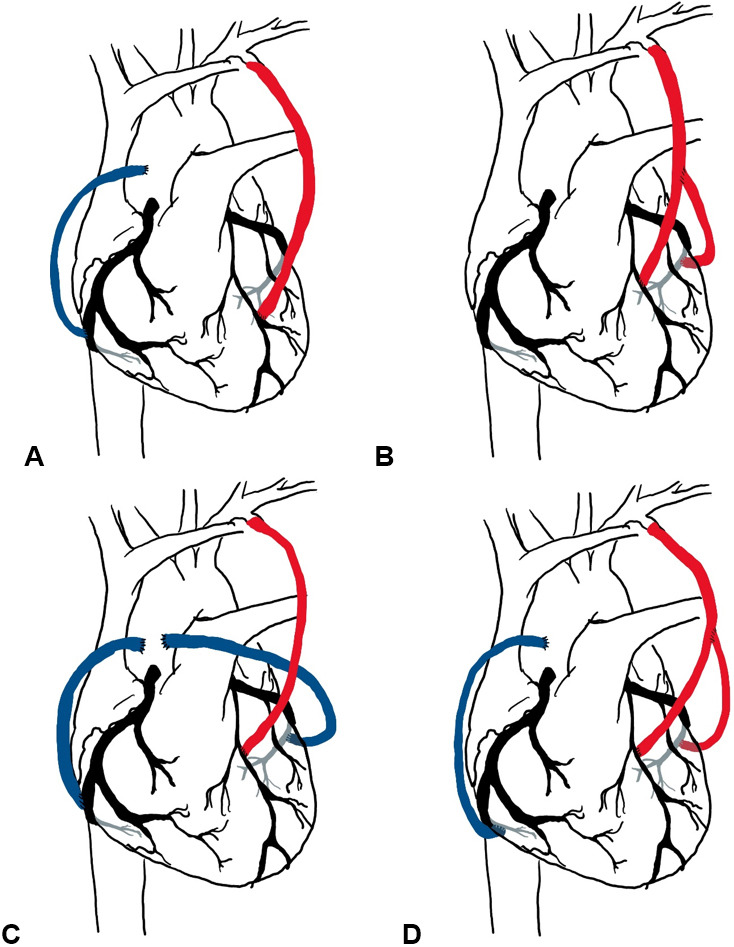
**Revascularization technique**. In the sITA group, the left ITA was harvested and anastomosed to the left anterior descending coronary (LAD) artery. One or two saphenous veins (VSM) were used to revascularized other coronary arteries (Figures A and C). In the dITA group, the left ITA was anastomosed to the LAD artery, and the right ITA was anastomosed as a T or Y graft usually to the circumflex artery system. Additionally, saphenous vein was sometimes used (Figure B and D).

In the sITA group, the entire length of the left ITA was harvested and anastomosed to the left anterior descending coronary (LAD) artery. One or two saphenous veins (VSM) were used to revascularize other coronary arteries (Fig. 2A,C). In the dITA group, the entire length of the left ITA (LITA) was harvested and anastomosed to the LAD artery, and a segment of variable length of the right ITA (RITA) was harvested and anastomosed as a T or Y graft usually to the circumflex artery system (Fig. 2B). In some cases additional saphenous veins were used (Fig. 2D).

Normothermia or mild hypothermia (34 °C) was induced. Custodiol® (histidine-tryptophan-ketoglutarate cardioplegia, prepared by the Department of Pharmacology, University hospital of Mainz, Mainz, Germany) was the solution of choice used for cardioplegia [10]. In this regard, it is worth mentioning that our institution switched to Calafiore cardioplegia, as the standard of choice for selective CABG surgery, starting 2021. Anastomoses were conducted using Polypropylene sutures (Ethicon Prolene Suture 7-0 or 8-0).

### 2.3 Outcome Measures

Postoperative 30-day mortality was the main outcome. Further outcomes included: (I) *intraoperative parameters* (cardiopulmonary bypass time; skin-to-skin operative time; volume of blood transfused, number of anastomoses carried out) and (II) *postoperative parameters* (ventilation time; length of in-hospital stay; intra-aortic balloon pump application; postoperative cardiopulmonary resuscitation or myocardial infarction; sternal wound healing disorders, need for re-thoracotomy and its reason). Ventilation time included both intraoperative and ICU ventilation. Re-thoracotomy included every chest opening conducted within 30 days following the main surgical intervention, for reasons such as bleeding/tamponade, graft complication (including twisting, occlusion, or other compromise of the bypass graft), sternal instability, mediastinitis, and explorative re-thoracotomy in the event of low cardiac output or cardiopulmonary resuscitation for unknown reasons. Relevant wound healing disorders included only those that required a surgical treatment, even if minor.

### 2.4 Statistical Analysis

Statistical analyses were performed using Microsoft Excel 2016 (Microsoft, Redmond, WA, USA), XLSTAT statistical and data analysis solution (Addinsoft, New York, NY, USA) and GraphPad Prism 9.2.0 (GraphPad, San Diego, CA, USA).

Categorical variables were presented by frequencies and percentages. Quantitative variables, which follow the Gaussian distribution, were described by their arithmetic means and standard deviation. Non-Gaussian quantitative variables were reported by the Median and 1st and 3rd quantile. Due to the high volume of data, normality was determined using both the Kolmogorov-Smirnov test and by observing the histogram. We chose alpha value of 0.05 for the 2-tailed *p* value.

Data was prepared manually and in an automated fashion by testing them for credibility and consistency. Variables that were found to be faulty or subject to surgeon subjectivity were removed. A primary round of statistical analysis provided a general view of the results before the matching. Categorical variables were compared using chi-square test and continuous variables were compared using the Student’s *t*-test when the distribution was Gaussian or the Mann-Whitney test otherwise.

As the baseline characteristics of the dITA can differ systematically from the sITA, we used the propensity score matching technique to identify two similar paired subgroups, as described by Austin [11]. To calculate the propensity scores for individual cases, a multiple logistic regression model was used. The following baseline-measured variables were considered relevant with regard to decide between sITA vs dITA: age and gender; body mass index (BMI); diabetes mellitus (DM); chronic obstructive pulmonary disease (COPD); peripheral artery disease; history of past myocardial infarction; creatinine clearance (calculated by the *Cockroft-Gault Method * [12]); ejection fraction (EF); New York Heart Association Functional Classification (NYHA); Euroscore II (calculated as described by Nashef *et al*. [13]) and the number of the treated coronary vessels. Greedy matching was conducted one-to-one between the dITA and sITA cases, based on the logit of the propensity scores, by finding the nearest neighbor within a specified caliper width of 0.2 standard deviations, and without replacement. Standardized differences were calculated to assess the balance in the baseline variables after the matching. To estimate the effect of sITA vs dITA, appropriate methods for analyzing paired, e.g., such McNemar’s test for binomial outcome, paired samples *t*-test and the Wilcoxon signed rank test were used.

## 3. Results 

During the period of the study, 1855 CABG operations that meet the study criteria were found. In patients undergoing sITA (n = 1114), 614 underwent a combination of LITA & 1× saphenous vein graft (VSM), while 500 underwent LITA & 2×VSM. In patients undergoing dITA (n = 741), 329 underwent LITA & RITA, and 412 underwent LITA & RITA & 1×VSM.

Comparing the unmatched sITA and dITA groups (Table 1A), there were slight differences in the patient characteristics age, BMI, past myocardial infarction and in NYHA class. These differences may suggest that dITA was selected for less complicated CABG surgeries.

**Table 1.** **Patient characteristics**.

		(A) unmatched groups			(B) matched groups			
		Single ITA	Double ITA	*p* value	Single ITA	Double ITA	*p* value	Standardized differences
		[N = 1114]	[N = 741]		[N = 519]	[N = 519]		
Gender (f/m)		356/758	197/544	0.013	142/377	154/365	0.450	0.051
Mean age ± SD		79.2 ± 3.0	78.8 ± 2.8	0.004	79.2 ± 2.9	79.1 ± 3.0	0.708	0.133
Mean BMI ± SD		27.6 ± 4.3	27.2 ± 4.1	0.04	27.4	27.4	0.729	0.076
Diabetes mellitus (%)		417 (37.4)	247 (33.3)	0.071	179 (34.5)	176 (33.9)	0.896	0.012
Chronic pulmonary disease (%)		146 (13.1)	77 (10.4)	0.078	66 (12.7)	64 (12.3)	0.925	0.011
Peripheral artery disease (%)		219 (19.7)	139 (18.8)	0.63	109 (21.0)	98 (18.9)	0.484	0.013
Past myocardial infarction (%)		400 (35.9)	212 (28.6)	0.001	172 (33.1)	160 (30.8)	0.462	0.049
	Within last 3 months (%)	200 (18.0)	78 (10.5)	<0.001	76 (14.6)	57 (11.0)	0.078	0.109
Neural impairment (%)		80 (7.2)	46 (6.2)	0.414	39 (7.5)	38 (7.3)	0.906	0.007
Creatinine clearance		59.7 ± 19.9	62.1 ± 19.5	0.01	60.2 ± 20.9	60.9 ± 19.2	0.582	0.117
LVEF category								
	Reduced (30–49%)	256 (23.0)	180 (24.3)	0.539	129 (24.9)	137 (26.4)	0.885	0.037
	Poor (<30%)	50 (4.5)	26 (3.5)	0.339	30 (5.8)	22 (4.2)	0.319	0.074
NYHA								
	III (%)	390 (35.0)	338 (45.6)	<0.0001	248 (47.8)	238 (45.9)	0.576	0.019
	IV (%)	79 (7.1)	72 (9.7)	0.046	55 (10.6)	50 (9.6)	0.681	0.021
Urgency (elective/urgent)		365/376	561/553	0.642	250/269	254/265	0.852	0.015
Median of Euroscore II		2.94%	2.83%	0.301	3.06%	2.88%	0.161	0.237

BMI, body mass index; ITA, internal thoracic artery; LVEF, left ventricle ejection fraction; NYHA, New York Heart Association Functional Classification; SD, standard deviation.

Five hundred and nineteen (519) pairs of LITA-dITA were matched. Details of the logistic regression model are available in Table 2. The paired groups were very similar in their characteristics (Table 1B).

**Table 2.** **Logistic regression model for calculating the propensity score**.

				Source	Value	*p*	OR
Goodness of fit statistics:			*p*	Intercept	422.34	<0.0001	
$R^{2}$ (McFadden)			0.204				
$R^{2}$ (Cox and Snell)			0.240	Age	0.08	0.000	1.08
$R^{2}$ (Nagelkerke)			0.324	Gender (male)	0.01	0.966	1.01
				Year of surgery	–0.21	<0.0001	0.81
				BMI	0.04	0.003	1.04
				DM	0.09	0.514	1.09
Test of the null hypothesis	χ ^2^	df	*p*	COPD	0.22	0.201	1.25
–2 Log (Likelihood)	15	508	<0.0001	Extracard. art. pathology	–0.11	0.528	0.89
Score	15	448	<0.0001	Neuro dysfunction	0.37	0.087	1.45
Wald	15	352	<0.0001	Creatinine clearance	0.00	0.878	1.00
				Past myocardial infarction	0.31	0.013	1.37
Hosmer-Lemeshow Statistic			0.081	Logit of ES II	0.22	0.275	1.25
				NYHA-class	–0.24	0.002	0.78
				LVEF-class	0.02	0.861	1.02
Area under the curve			0.792	Count of grafts	–0.55	<0.0001	0.58
				Harvesting technique (skeletonized)	–0.53	<0.0001	0.59

BMI, body mass index; DM, Diabetes mellitus; COPD, chronic obstructive pulmonary disease; LVEF, left ventricle ejection fraction; NYHA, New York Heart Association Functional Classification; OR, odd ratio.

**Fig. 3.**
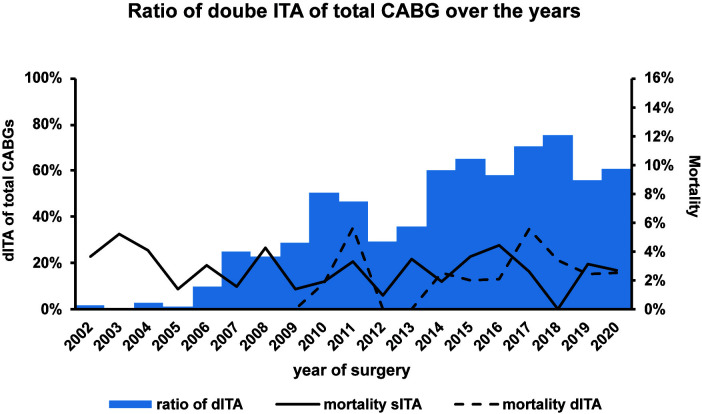
**The change of the ratio of performed dITA over the ratio**. There was a tendency to perform more dITA in the recent years. Mortality for both sITA and dITA was relatively steady, except for a slight elevation in early years. The “zero” mortality of dITA in early years is a result of a low total number (less than 20 dITA for before 2007).

Year of surgery was a significant predictor for performing double ITA, indicating higher tendency for performing double ITA in the more recent years in our department (Fig. 3). Mortality however was not affected by this tendency.

Significant differences were seen in median skin-to-skin operative time (181 vs 205 minutes; *p *< 0.0001), in ventilation time (10 vs 9 hours; *p* = 0.020) and in the incidence of wound healing disorders, which was significantly higher when utilizing dITA (3.3 vs 6.9%; *p* = 0.011). There was no significant difference in the incidence of sternal wound healing disorders when comparing the skeletonized against the pedicled technique (4.2 vs 6.2%, *p* = 0.135. Calculation for the total number in both the matched sITA and dITA groups). The rest of the outcome parameters were similar. Table 3 lists the detailed results (Tables 3,4).

**Table 3.** **Intraoperative parameters**.

	Unmatched groups			Matched groups		
	Single ITA	Double ITA	*p* value	Single ITA	Double ITA	*p* value
	[N = 1114]	[N = 741]		[N = 519]	[N = 519]	
Harvesting technique	772p/342s	287p/454s	<0.0001	241p/278s	243p/276s	0.945
Median CPB time [IQR]	72 [57–88]	77 [63–96]	<0.0001	75 [60–92]	74 [61–93]	0.860
Median skin-to-skin time [IQR]	174 [147–207]	210 [145–243]	<0.0001	181 [155–217]	205 [172–239]	<0.0001
Mean of anastomoses per surgery	2.70	2.66	0.538	2.67	2.57	0.614
Blood transfused per surgery (mL)	323	349	0.557	338	346	0.471
Bypass configuration			<0.0001			0.059
2 grafts (%)	614 (55.1)	329 (44.4)		288 (55.5)	283 (54.5)	
3 grafts (%)	500 (44.9)	412 (55.6)		231 (44.5)	236 (45.5)	

CPB, cardio-pulmonary bypass; IQR, interquartile range; ITA, internal thoracic artery.

**Table 4.** **Postoperative outcome**.

	Unmatched groups			Matched groups		
	Single ITA	Double ITA	*p* value	Single ITA	Double ITA	*p* value
	[N = 1114]	[N = 741]		[N = 519]	[N = 519]	
Median of ventilation time in hours [IQR]	10 [4–16]	9 [6–13]	0.054	10 [7–15]	9 [6–13]	0.020
Median of in-hospital stay in days [IQR]	9 [7–13]	10 [1–13]	0.320	9 [7–12]	10 [7–13]	0.203
Use of IABP (%)	19 (1.7)	19 (2.6)	0.089	12 (2.3)	14 (2.7)	0.845
CPR (%)	42 (3.8)	21 (2.8)	0.699	18 (3.5)	17 (3.3)	0.863
Myocardial infarction (%)	15 (1.3)	7 (0.9)	0.494	3 (0.6)	6 (1.2)	0.505
Sternal wound healing disorders (%)	29 (2.6)	51 (6.9)	<0.0001	17 (3.3)	36 (6.9)	0.011
skeletonized	8 (2.3)	27 (5.9)	<0.0001	6 (2.2)	17 (6.2)	0.037
pedicled	21 (2.7)	24 (8.4)	0.021	11 (4.6)	19 (7.8)	0.201
Tracheotomy (%)	13 (1.2)	12 (1.6)	0.053	4 (0.8)	10 (1.9)	0.018
Cerebrovascular events (%)	46 (4.1)	22 (3.0)	0.096	19 (3.7)	19 (3.7)	0.871
Re-thoracotomy	69 (6.2)	40 (5.4)	0.116	24 (4.6)	32 (6.2)	0.340
Bleeding or tamponade (%)	45 (4.0)	16 (2.2)	0.033	16 (3.1)	15 (2.9)	0.855
Graft problems (%)	6 (0.5)	6 (0.8)	0.559	2 (0.4)	6 (1.2)	0.286
Sternal instability or infection (%)	13 (1.2)	15 (2.0)	0.173	3 (0.6)	11 (2.1)	0.061
Low cardiac output or CPR (%)	1 (0.1)	2 (0.3)	0.567	0 (0.0)	0 (0.0)	-
30 days Mortality	32 (2.9)	18 (2.4)	0.661	17 (3.3)	15 (2.9)	0.859

CPR, cardio-pulmonary resuscitation; IABP, intra-aortal balloon pump; IQR, interquartile range; ITA, internal thoracic artery.

## 4. Discussion

Shortly after the introduction of the use of ITA as a graft in CABG procedures in the 60s [14], it became clear that this practice is superior to utilizing vein grafts, e.g., the saphenous vein [15]. The saphenous vein graft, which is still widely used in bypass surgery due to its availability, is prone to progressive intimal hypertrophy and occlusion, leading to impaired long-term graft patency, which usually presents after the fifth postoperative year, but sometimes much earlier [16, 17, 18]. Studies in the 1980s and 1990s led to widespread utilization of the ITAs in CABG surgery. Very few other arteries in the human body can be harvested as easily and with minimal potential collateral damage as the ITAs. Anastomosing the LITA to the LAD artery became a standard surgical approach [19]. The logical next step was to harvest the ITA bilaterally, assuming that superior patency would correlate with improved long-term results.

Many observational studies emerged and reported better results for dITA compared to LITA in different patient groups [20, 21, 22]. The general impression of the superiority of dITA was questioned with the publishing of the primary and final results of the only available randomized controlled study (RCT) in this regard; *the Arterial Revascularization Trial (ART) * [6, 20, 23]. The ART investigator had conducted the largest multicenter randomized trial of bilateral versus single ITA grafts and followed 3102 patients in seven countries for up to 20 years. Surprisingly, they found no advantage of utilizing dITA compared to sITA in regard to mortality or the rate of cardiovascular events at both 5 years and 10 years of follow-up (5-years mortality 8.7% in dITA and 8.4% in sITA). However, they found higher rates of sternal wound complications in the dITA group (3.5% vs 1.9% in sITA). Multiple limitations may possibly have influenced the results, masking better results for dITA [7]. The discrepancy in the results between these studies may be partially attributed to the heterogeneity of techniques of anastomosing and reconstruction, ranging from in situ dITA to complex Y-anastomoses (Fig. 2). In addition, the classical LITA-LAD bypass is often protected by high blood flow, which may play a role in the better LITA results.

A long-term survival advantage in elderly is hard to prove as the life-expectancy shortens. Few groups have investigated this, and there has been no conclusive consensus on the matter. Navia *et al*. [24] conducted a retrospective analysis of 243 matched sITA-dITA pairs of patients >70 years of age and found survival advantage for dITA after 10 years of follow-up (66% vs 53.0%, *p* = 0.022). Medalion *et al*. [25] also found a survival advantage for dITA, but their study was unmatched and their compared group was not homogeneous. In contrast, both Mohammadi *et al*. [26] and Benedetto *et al*. [27] found no survival advantage in patients older than 65 years. Other studies included only small numbers of patients and have not been conclusive. In conclusion, the potential cut-off age for possible survival benefit of dITA remains to be determined. In this context, one should note the variability in the age cutoff defining the elderly groups in different observational studies. We chose a 75 year cutoff based on national demographic trend, as reported by the German Association for Thorax, Heart and Vascular surgery (DGTHG) [28], and based on our own statistics, that showed almost 30% of CABG was conducted on patients 75 years and older (Fig. 1).

Our study confirms the safety of harvesting both ITAs in the elderly without increasing the perioperative mortality but indicates a significant increase in the operation time and the incidence of sternal wound healing disorders. This suggests that the practice can be justified only if a significant long-term advantage is to be expected, which as discussed before, remains uncertain. Wound healing disorders have a severe negative effect on patients’ quality of life and can even impact short-term survival rate beyond the first 30 days. Nevertheless, sternal wound healing disorders can be avoided by careful patient selection, obesity, female gender and diabetes mellitus adversely influence the outcome. In addition, harvesting the ITA in a skeletonized way significantly reduces wound healing disorders [29, 30]. However, our study failed to establish such a correlation. Also, our study failed to find a statistically significant difference in the rate of sternal complications (0.6% vs 2.1%, *p = *0.06). A bigger sample size is most-likely necessary to prove this as the total number of sternal complications (14 for both matched groups) was too low to avoid type II error.

Reviewing all the pros and cons for harvesting both ITAs in elderly patients that were reported by our study and others, we believe that harvesting both ITAs is safe and can be done in selected patients despite their age, whose overall life expectancy are good and whose risk factors for wound healing disorders are mild and when the viability of the saphenous vein is questionable.

**Fig. 4.**
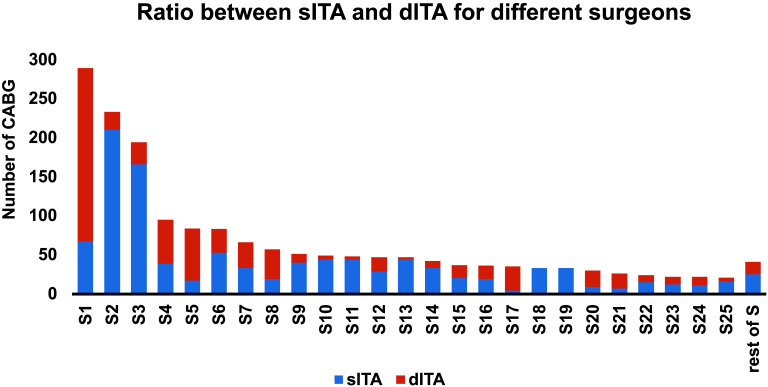
**The ratio between sITA and dITA for the different surgeons in the study**. The decision for sITA vs dITA was not standardized and hence individual differences were noticed between the surgeons.

Finally, study limitations include: (I) This study is prone to the usual limitation of a retrospective analysis, such as the quality of the data and their heterogeneity. Operative urgency for example, lacked a clear definition and was surgeon dependent, hence, it was not included in the propensity score matching calculation. (II) Considering the time-frame of almost 20 years, changes in surgical practice and patient care could have affected the results. More dITA and skeletonized harvesting were performed in the recent years (Fig. 3). The time factor can influence other variables in an unpredictable way. However, matching should minimize such effects. (III) Being a single-center study, there is risk of outcome data being affected by local experience in CABG practice; and (IV) data on exact classifications of wound healing disorders were not available to us, which is why we only considered wounds that have been treated surgically. (V) The decision for sITA or dITA was not standardized and hence remains subjective and dependent on the surgeon himself. Fig. 4 shows a clear trend for some surgeons to favor LITA over sITA. However, this is partially also due to the time effect as early surgeons favored sITA (Fig. 3). (VI) Comparing the unmatched sITA and dITA groups (Table 1), there were slight differences in the patient characteristics age, BMI, past myocardial infarction and in NYHA class. These differences may suggest that dITA was selected for less complicated CABG. However, propensity score matching should have compensated for this effect. (VII) Lastly, there are some limitations to the statistical methods that were used. Variables such as ventilation time and in-hospital stay were strongly skewed. While the median ventilation time was shorter in the double ITA group (9 h vs 10 h), the average was actually higher (24.8 vs 20.1 h). This skewness resulted from outliers from patients who had required significantly longer ventilation times (exceeding 2 months in some cases) and resulted in a positive Wilcoxon Signed Rank test (*p* = 0.02). The most significant prediction for longer ventilation time was re-thoracotomy (*p* = 0.004) and it was seen more often in the double ITA sample (6.2% vs 4.6%).

## 5. Conclusions

In conclusion, harvesting both ITA in elderly patients is safe and feasible. However, it is associated with increase in the incidence of sternal wound healing disorders. When done, attention is required to control other factors influencing the wound healing. Long-term benefit of dITA grafting still needs to be proven.

## Data Availability

The data related to this study are available on request from the corresponding author, RC.

## Abbreviations

ART, Arterial Revascularization Trail; CABG, coronary artery bypass graft; COPD, chronic obstructive pulmonary disease; EF, ejection fraction; ITA, internal thoracic artery; LAD, left descending coronary artery; NYHA, New York Heart Association; VSM, vena saphena magna.
